# Identification and profiling of growth-related microRNAs in Chinese perch (*Siniperca chuatsi*)

**DOI:** 10.1186/s12864-017-3851-y

**Published:** 2017-06-28

**Authors:** Jiagang Tu, Changxu Tian, Peiqi Zhao, Junxiao Sun, Min Wang, Qixue Fan, Yongchao Yuan

**Affiliations:** 1 College of Fisheries, Key Lab of Agricultural Animal Genetics, Breeding and Reproduction of Ministry of Education/Key Lab of Freshwater Animal Breeding, Ministry of Agriculture, Huazhong Agricultural University, Wuhan, Hubei 430070 China; 2 Freshwater Aquaculture Collaborative Innovation Center of Hubei Province, Wuhan, Hubei 430070 China

**Keywords:** MicroRNAs, Chinese perch, Growth, Deep sequencing, qRT-PCR

## Abstract

**Background:**

MicroRNAs (miRNAs) are endogenous small non-coding RNAs that play important roles in the regulation of diverse biological processes in eukaryotes. Chinese perch (*Siniperca chuatsi*) is one of the most economically important fish species widely cultured in China. Growth is an extremely important characteristic in fish. Individual differences in body size are common in *Siniperca chuatsi,* which significantly influence the aquaculture production of *Siniperca chuatsi*. However, the underline growth-related regulatory factors, such as miRNAs, are still unknown.

**Results:**

To investigate the growth-related miRNAs in *Siniperca chuatsi,* two RNA libraries from four growth-related tissues (brain, pituitary, liver, and muscle) of *Siniperca chuatsi* at 6-month stage with relatively high or low growth rates (big-size group or small-size group) were obtained and sequenced using Solexa sequencing. A total of 252 known miRNAs and 12 novel miRNAs were identified. The expression patterns of these miRNAs in big-size group and small-size group were compared, and the results showed that 31 known and 5 novel miRNAs were differently expressed (DE). Furthermore, to verify the Solexa sequencing, five DE miRNAs were randomly selected and quantified by quantitative reverse transcription polymerase chain reaction (qRT-PCR). The results showed that their expression patterns were consistent with those of Solexa sequencing. In addition, Gene Ontology (GO) and Kyoto Encyclopedia of Genes and Genomes (KEGG) enrichment analysis of target genes of DE miRNAs was performed. It showed that the target genes were involved in multiple biological processes including metabolic process, suggesting that metabolic process played an important role in growth of fish.

**Conclusions:**

*Siniperca chuatsi* is a popular and economically important species in aquaculture. In this study, miRNAs in *Siniperca chuatsi* with different growth rates were identified, and their expression profiles were compared. The data provides the first large-scale miRNA profiles related to growth of *Siniperca chuatsi*, which is expected to contribute to a better understanding of the role of miRNAs in regulating the biological processes of growth and possibly useful for *Siniperca chuatsi* breeding.

**Electronic supplementary material:**

The online version of this article (doi:10.1186/s12864-017-3851-y) contains supplementary material, which is available to authorized users.

## Background

The Chinese perch (*Siniperca chuatsi*), with delicious flesh, is a popular and economically important fish species in many Asian countries. However, the cultured population of *Siniperca chuatsi* gradually exhibits growth depression, early sexual maturity, and disease susceptibility. Therefore, molecular techniques are needed to unveil these characteristics. Although the growth-related genes or single nucleotide polymorphisms of growth hormone gene of *Siniperca chuatsi* have been investigated [1, 2], the regulatory role of small RNAs, such as microRNA, on the growth of *Siniperca chuatsi* is still elusive.

**Table 1:** Primers for qRT-PCR used in this study

Name	Forward primers (5′-3′)	Backward primers (5′-3′)
miR-10d-5p	TACCCTGTAGAACCGAATGTGTG	Universal primer^a^
miR-126b-5p	CATTATTACTTTTGGTACGCG	As above
miR-133a-3p	TTTGGTCCCCTTCAACCAGCTG	As above
miR-142a-3p	TGTAGTGTTTCCTACTTTATGGA	As above
miR-202-3p	AGAGGCATAGGGCATGGGAAAA	As above
U6	CTCGCTTCGGCAGCACA	AACGCTTCACGAATTTGCGT

^a^: It was provided from All-in-One™miRNA qRT-PCR Detection Kit

**Table 2:** Overview of the Solexa sequencing data

	Small-size group	Big-size group
Raw reads	16,602,433	16,048,981
Clean reads	15,351,486	14,438,433
Unannotated reads	8,427,566	7,962,496
Mapped reads^a^	7,256,936	7,156,763

^a^: After the rRNA, scRNA, snRNA, snoRNA, tRNA, and repbase were identified from the clean reads, the left unannotated reads were aligned with the genome of zebrafish to determine the mapped reads

**Table 3:** Identification of DE miRNAs

Name	Small-size group^#^	Big-size group	FDR	Log_2_^Fold changea^*	Regulation
miR-1	87,650.34892	34,516.48053	0	−1.344474467	down
miR-10d-5p	5761.977097	12,698.06025	0	1.139972302	up
miR-126a-5p	292.0125674	667.1046915	0	1.191882585	up
miR-126b-5p	291.5991471	666.1259171	0	1.19180861	up
miR-133a-5p	661.7481589	228.3340937	0	−1.535136034	down
miR-133a-3p	5668.268487	2069.548635	0	−1.453591971	down
miR-133b-3p	369.5977847	111.4404609	0	−1.729682533	down
miR-137-5p	1.5159	0.1398	0.001586	−3.43873832	down
miR-137-3p	42.99571545	19.43566383	0	−1.1454833	down
miR-142a-5p	494.1751141	1439.917022	0	1.54289143	up
miR-142a-3p	403.7738662	943.8182075	0	1.224961334	up
miR-144-3p	388.7529272	1023.798061	0	1.39700589	up
miR-150	0.2756	1.3982	0.007654	2.342922947	up
miR-153b-5p	1.7915	0.6991	0.017438	−1.357597303	down
miR-155	91.22808855	201.767359	0	1.14514292	up
miR-182-3p	0.5512	2.0974	0.003435	1.927954234	up
miR-196a-5p	162.6120007	25.30831045	0	−2.6837512	down
miR-196b	3.445169507	0.279649839	0.00000429	−3.6231515	down
miR-19b-5p	1.240261023	2.516848553	0.02231009	1.02090146	up
miR-200b-5p	23.01373231	9.368269615	0	−1.2966338	down
miR-202-5p	2068.204159	485.8915957	0.000002	−2.0896722	down
miR-202-3p	20.2575967	2.516848553	0.000026	−3.0088008	down
miR-203a-3p	348.099927	132.4141989	0	−1.3944436	down
miR-203b-5p	4.547623749	1.118599357	0.000052	−2.0234111	down
miR-203b-3p	1.515874583	0.419474759	0.01697204	−1.8534319	down
miR-205-5p	75.65592238	19.85513859	0	−1.929943	down
miR-206-3p	8521.006645	2130.23265	0	−2.0000128	down
miR-26a-2-3p	0.689033901	2.516848553	0.00302765	1.86901469	up
miR-460-3p	0.413420341	1.118599357	0.04857661	1.43608394	up
miR-499-5p	252.1864079	125.4229529	0	−1.0076885	down
miR-499-3p	1.102454242	0.279649839	0.02966909	−1.9793424	down
Novel-miR-1	1.3781	0.1398	0.00453429	−3.3012443	down
Novel-miR-3	1.2403	0.4195	0.0357451	−1.5639464	down
Novel-miR-8	1.5159	0.4195	0.01697204	−1.8534319	down
Novel-miR-9	1.5159	0.5593	0.02860011	−1.4384804	down
Novel-miR-11	1.1024	0	0.00339448	−23.394275	down

^*^: Fold change indicates the TPM values of each miRNA from big-size group were compared to those from small-size group

^#^: The values from small-size group and big-size group indicate transcript per million (TPM)

MicroRNAs (miRNAs) are a class of endogenous, non-coding, ~22-nt RNA molecules, which exist in a wide range of invertebrates and vertebrates [3]. It has been well known that miRNAs are involved in various biological processes in eukaryotes, including cellular proliferation, development, differentiation, metabolism, oncogenesis, and apoptosis [3–6]. They negatively regulate gene expression through binding to the 3′ untranslated regions (UTRs), 5’UTRs, or the coding regions of target mRNA [7–9]. Since the first discovery of the miRNAs lin-4 and let-7 in *Caenorhabditis elegans* in 1993 [10], numerous miRNAs have been identified in mammals, plants, insects, worms, and viruses [11–14]. However, miRNA information is limited in fish species, with only 9 out of the 30,000 fish species present in miRBase [15–20].

High-throughput next-generation sequencing has become a powerful tool for transcriptomic and miRNA sequencing. MiRNAs have been identified in many fish species such as rainbow trout (*Oncorhynchus mykiss*) [21], Japanese flounder (*Paralichthys olivaceus*) [22], bighead carp (*Hypophthalmichthys nobilis*) [23], common carp (*Cyprinus carpio*) [24], and channel catfish (*Ictalurus punctatus*) [25]. However, the information on microRNA in *Siniperca chuatsi* is still unknown. Growth is one of the most important characteristics in fish. Growth-related miRNAs in blunt snout bream have previously been investigated through analyzing miRNA expression profiles from four growth-related tissues including brain, pituitary, liver, and muscle of big-size fish with relatively high growth rate and small-size fish with relatively low growth rate [26]. Individual differences in body size are common in *Siniperca chuatsi,* which significantly influence the aquaculture production of *Siniperca chuatsi*. In this study, we performed small RNA sequencing for the RNAs extracted from four growth-related tissues (brain, pituitary, liver, and muscle) of *Siniperca chuatsi* using individuals with relatively high and low growth rates (big-size group and small-size group). The miRNAs in the two groups of *Siniperca chuatsi* were identified and their expressions were compared. The DE miRNAs were further analyzed, and GO and KEGG enrichment analysis for the predicted target genes were performed. To our knowledge, our data provides the first survey of growth-related miRNAs in *Siniperca chuatsi,* which will contribute to a better understanding of the role of miRNAs in regulating the growth in fish.

## Methods

### Animals and tissue collection

All fish from the same family were fed with live mrigal *Cirrhinus mrigala*. All experimental procedures involving fish were approved by the institution of animal care and use committee of Huazhong Agricultural University. 60 fish with extreme phenotypes were selected at 3-, 6-, or 12-month stages, respectively: three small-size groups (S_3,_ S_6_ and S_12_) and three big-size groups (B_3,_ B_6_ and B_12_) from 1500 individuals [1]. Six fish in big-size or small-size groups at 6-month stage were euthanized with tricaine methanesulfonate (MS-222) at 100 mg/L. Four tissue samples including brain, pituitary, liver, and muscle of the fish were immediately frozen in liquid nitrogen upon surgical resection to avoid degradation and stored at −80 °C prior for RNA isolation.

### Small RNA isolation and cDNA library construction

Total RNAs were extracted from the tissue samples including brain, pituitary, liver, and muscle from big-size group and small-size group of fish at 6-month stage using Trizol reagent (TaKaRa, Dalian, China) according to the manufacturer’s protocol. RNA quality and quantity was measured using the NanoDrop 2000 (Thermo Scientific, Wilmington, DE, USA) and then standardized to 500 ng/μl. Equal volumes of RNAs from different tissues of fish in the same group were combined into one pool. Small RNAs of 18–30 nt in length were first isolated from the total RNAs by size fractionation, then were ligated with 3′-RNA adapters, and finally created cDNA libraries using reverse transcription PCR. The amplified cDNA constructs were purified and sequenced by Illumina/Solexa technology (BMK, Beijing, China).

### Sequence data analysis

The raw reads from Solexa sequencing were processed by evaluating sequencing quality. After eliminating low quality reads, adaptor sequences, and redundancy contaminant from raw reads, the clean reads were obtained. The clean reads with a length of 18–30 nt were blasted against the Rfam database and the GenBank noncoding RNA database to annotate rRNA, tRNA, snRNA, repeat associate small RNA, and other ncRNA sequences. Since the whole-genome sequence of *Siniperca chuatsi* is unknown, the remaining reads were used to map to the zebrafish (*Danio rerio*) genome. The mapped reads were then aligned with zebrafish miRNAs and the sequences with perfect match were identified as known miRNAs. The sequences that are not identical to the zebrafish miRNAs were used for novel miRNAs prediction using the Short Oligonucleotide Analysis Package (SOAP) software [27].

### Analysis of DE miRNAs

To identify DE miRNAs between the big-size group and small-size group, the expression of miRNA in the two groups were normalized to obtain the expression of transcripts per million as described before [26]. The false discovery rate (FDR) < 0.05 and |log_2_^(fold change)^| > 1 was set as the threshold for significantly differential expression.

### qRT-PCR analysis of five DE miRNAs

Five randomly selected miRNAs were detected by qRT-PCR using the same RNA samples used for the construction of the small RNA libraries. The primers for the detection of these miRNAs were listed in Table 1. Quantitative PCR was performed using LightCycler® 480 Real-time PCR Instrument (Roche, Swiss) with 20 μl PCR reaction mixture containing 2 μl diluted cDNA, 400 nM of each primer, and 10 μl of qPCR Mix. Reactions were incubated in a 96-well optical plate (Roche, Swiss) at 95 °C for 10 min, followed by 45 cycles of 95 °C for 10 s, 58 °C for 10 s, and 72 °C for 10 s, and ended with 95 °C at 5 °C/s calefactive velocity to make the melt curve. All expression levels were normalized to the U6 gene [28]. Amplification results were analyzed using 2^-ΔΔCt^ method, which achieve results for relative quantification. Ct represents the threshold cycle.

### Target gene prediction and analysis

Considering that the genome information of *Siniperca chuatsi* is unavailable, we selected the zebrafish genome to predict the target genes with the strategy described previously [26]. GO and KEGG enrichment analysis were performed on the predicted target genes.

## Results and discussion

### Morphological characteristics

**Fig. 1:**
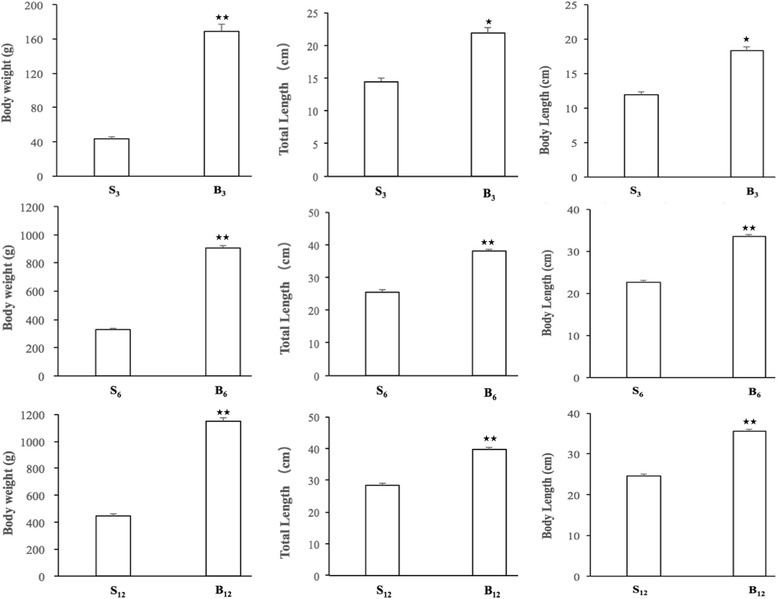
The morphological characteristics of big-size and small-size *Siniperca chuatsi* at 3-, 6- and 12-month stages. S_3_, S_6_, and S_12_ indicate small-size fish at 3-, 6-, and 12-month stages, respectively; B_3_, B_6_, and B_12_ indicate big-size fish at 3-, 6-, and 12-month stages, respectively. Total length means the length from the rostral tip of jaw to the caudal tip of the expanded tail, and body length refers to the length from the rostral tip of jaw to the caudal end of last lateral line scale. Data are mean ± S.D. (*n* = 30). The asterisks * and ** respectively indicated statistically significant differences between big-size group and small-size group (*: *p* < 0.05; **: *p* < 0.01)

Individual differences in growth are common in fish but little is known about its genetic control [29]. In the present study, we found that the morphological characteristics including body weight, total length, and body length of big-size groups at 3-, 6-, and 12-month stages (B_3,_ B_6,_and B_12_) were significantly higher than those of small-size groups (S_3,_ S_6,_ and S_12_), respectively (Fig. 1). Our previous study has revealed genes potentially influencing body size of *Siniperca chuatsi* at 6-month stage by transcriptome sequencing of mRNA libraries from big-size and small-size fish, and identified several up-regulated growth factors (TGF-b1, TGF-b1r, PDGF and VEGFR2) and key genes (Ras, RasGFR, RasGRP, PKC and PI3K) in the PI3K-Akt and/or Ras-MAPK pathways [1]. However, the miRNAs involved in the growth of *Siniperca chuatsi* have never been investigated.

### Overview of Solexa sequencing data

**Fig. 2:**
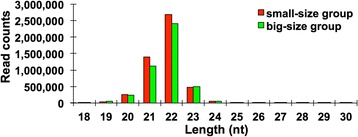
Length distribution of small RNAs derived from *Siniperca chuatsi.* The small RNAs from small-size and big-size groups with length between 18 and 30 nt are analyzed

**Fig. 3:**
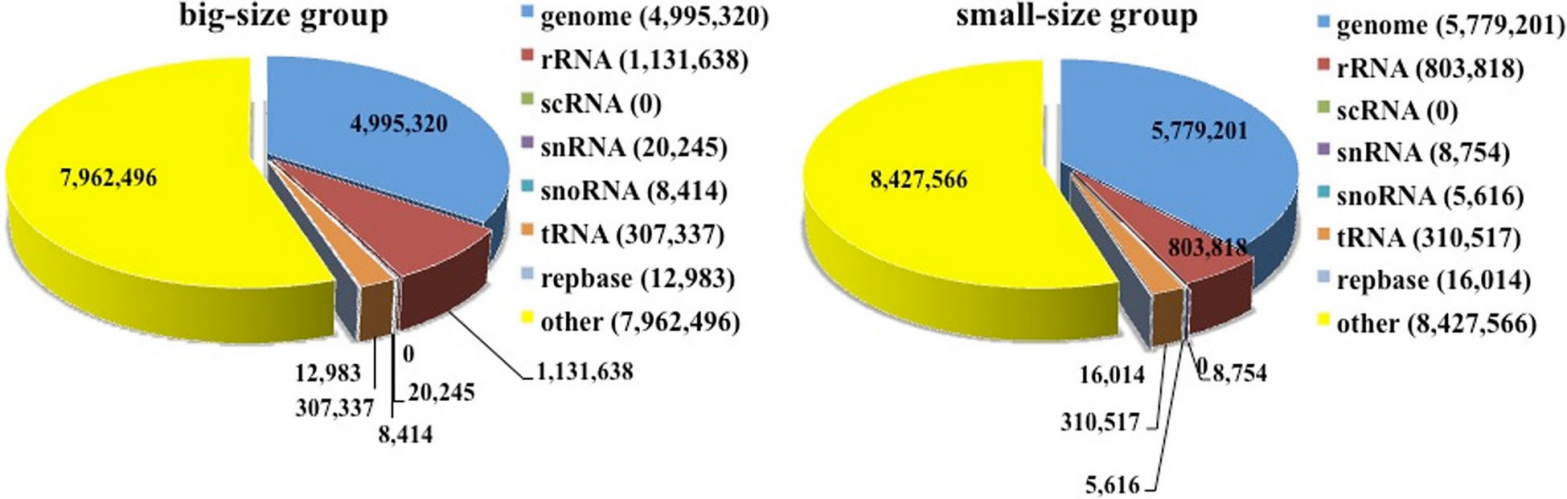
Annotation of small RNAs derived from Solexa sequencing of *Siniperca chuatsi.* The small RNAs from the big-size and small-size groups of *Siniperca chuatsi* were annotated after comparing with the NCBI GenBank and RFam database

To identify the growth-related miRNAs in *Siniperca chuatsi*, two small RNA libraries were constructed from four growth-related tissue samples (brain, pituitary, liver, and muscle) of small-size group and big-size group of *Siniperca chuatsi* at 6-month stage. High-throughput Solexa sequencing was then performed on the small RNA libraries. A total of 16,602,433 and 16,048,981 raw reads were obtained from small-size group and big-size group, respectively (Table 2). After removal of the adapters, junk reads, and reads with length < 18 nt or >30 nt, 15,351,486 and 14,438,433 clean reads were obtained (Table 2). Length distributions of the clean reads revealed that the majority of the reads were about 21–23 nucleotides and the 22-nt reads were the most abundant (Fig. 2). After comparing the clean reads with the NCBI GenBank and Rfam database, the reads of rRNA, tRNA, scRNA, snRNA, snoRNA and repeat-associated small RNAs were annotated (Fig. 3). The remaining unannotated reads (8,427,566 for small-size group and 7,962,496 for big-size group, respectively) were tried to map with the genome of zebrafish. We found that 86.1% and 89.9% (7,256,936 and 7,156,763) of the unannotated reads from small-size group and big-size group were mapped to the genome of zebrafish, respectively (Table 2).

### Identification of known and prediction of novel miRNAs in *Siniperca chuatsi*

Teleost is one of the most abundant species in vertebrate. However, only a total of 1250 miRNAs have been identified in 8 representative teleost species up to 2014 [15]. To identity the known miRNAs in *Siniperca chuatsi*, the clean reads were aligned with the zebrafish miRNAs in miRBase 20.0. A total of 252 known miRNAs were identified. Among them, 234 miRNAs were identified in both small-size group and big-size group. For the left 18 miRNAs, 9 miRNAs were expressed in small-size group, while the other 9 miRNAs were expressed in big-size group (Additional file 1). The expression levels of each miRNA in the two groups were calculated and presented as transcript per million (TPM) (Additional file 1). We found that the expression level of these miRNAs varied significantly, indicating that not only highly expressed but also lowly expressed miRNAs were detected by Solexa sequencing. Of the 252 miRNAs, the most abundant three miRNAs were miR-1, miR-22a-3P, and miR-21, with the TPM value higher than 80,000 (Additional file 1).

The small RNAs that could not be aligned with the known zebrafish miRNAs were subjected to novel miRNA prediction. According to the criteria for miRNA prediction as previously [26], we finally obtained 12 novel miRNAs in *Siniperca chuatsi*. Among them, 11 miRNAs were found in both small-size group and big-size group. Interestingly, the frequencies of these novel miRNAs were much lower than those of known miRNAs (Table 3 and Additional file 1).

### DE miRNAs

The expression levels of the known and novel miRNAs in small-size group and big-size group were compared. Totally 31 known and 5 novel miRNAs were DE (FDR < 0.05 and |log_2_ (fold change)| > 1) when the expression level of the miRNAs in big-size group was compared to those in small-size group (Table 3). Of the 31 known miRNAs, 12 miRNAs were upregulated and 19 miRNAs were downregulated. These DE known miRNAs were then compared with previously reported potential growth-related miRNAs in other fish species. The DE miRNAs have been previously investigated in *Portunus trituberculatus* through comparing miRNA expression profiles between big-size fish and small-size fish. We found that the downregulation of miR-1 and upregulation of miR-150 were present in both *Portunus trituberculatus* and *Siniperca chuatsi* when the miRNAs in big-size group were compared to those in small-size group [30]. These data suggested that the downregulation of miR-1 and upregulation of miR-150 might be involved in growth regulation in fish species. MiR-203 has been reported to inhibit cell growth through regulation of G1/S transition by targeting Bmi-1 in myeloma cells [31]. Here, we found that three miR-203 isomiRs including miR-203a-3P, miR-203b-5P, and miR-203b-3P were downregulated (Table 3). Therefore, the downregulation of miR-203 might probably enhance the expression of Bim-1 and thus facilitate the cell growth in *Siniperca chuatsi*. In addition, miR-155 could promote cancer cell growth by repressing the tumor suppressor cyclin-D binding myb-like transcription fector 1 (DMTF1) [32]. In this study, we found that miR-155 was upregulated (Table 3), indicating that the upregulation of miR-155 might inhibit the expression of DMTF1 and thus promote the cell growth of *Siniperca chuatsi*. Although we identified several possible growth-related miRNAs here, it is still needed to be determined in the future.

In addition to the DE known miRNAs, we also identified 5 DE novel miRNAs from the 12 novel miRNAs, and all five novel miRNAs were downregulated (Table 3).

**Fig. 4:**
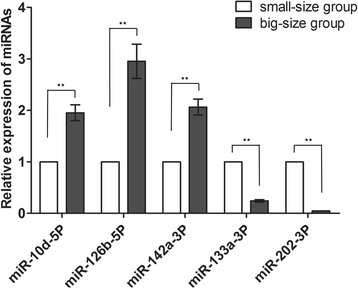
Expression analysis of five selected miRNAs by qRT-PCR. Five DE miRNAs were randomly selected to quantify their expression profiles using qRT-PCR. The expression level of small-size group was used as control and set 1. The U6 gene was used as internal gene. The asterisks * and ** respectively indicated statistically significant differences between big-size group and small-size group (*: *p* < 0.05; **: *p* < 0.01)

To validate the differential expression of the miRNAs between small-size group and big-size group, five randomly selected DE miRNAs including three upregulated miRNAs (miR-10d-5P, miR-126b-5P, miR-142a-3P) and two downregulated miRNAs (miR-133a-3P and miR-202-3P), were quantified using qRT-PCR. The mean normalized miRNA expression level was calculated and presented as the relative fold change. The results showed that all the five miRNAs exhibited a pattern consistent with the Solexa sequencing data (Fig. 4).

### Target gene prediction of DE miRNAs as well as GO and KEGG pathway enrichment

**Fig. 5:**
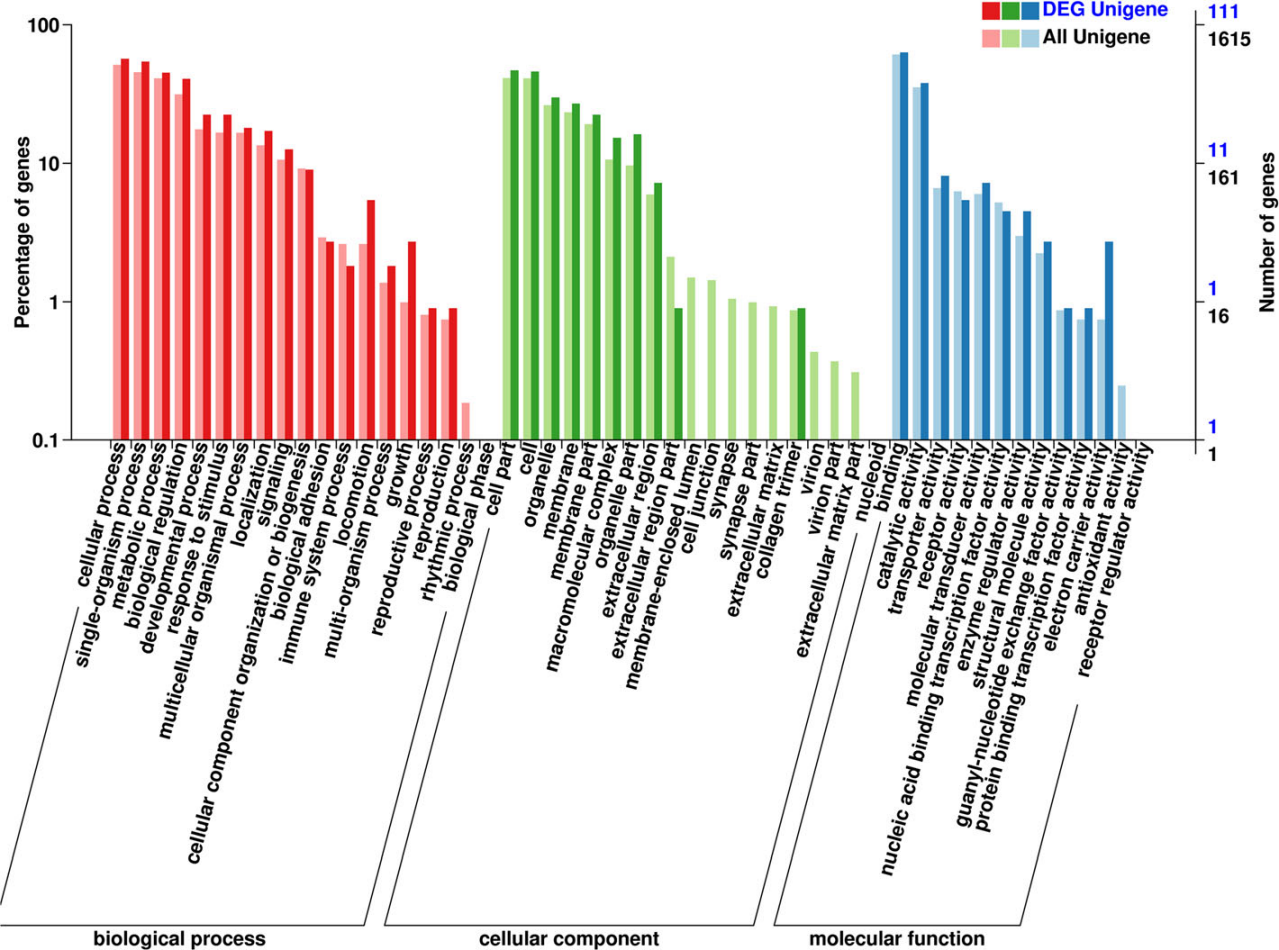
GO enrichment analysis of target genes of all miRNAs as well as DE miRNAs between big-size group and small-size group

Previous studies have revealed that the regulation of growth in fish is a complex regulatory process [29], and the potential growth-related genes have been investigated [33]. The analysis of target genes of DE miRNAs is important to illuminate the growth-related miRNAs. In this study, Miranda and RNAhybrid software were used to predict target genes according to zebrafish genome. A total of 172 target genes were identified for the 31 DE miRNAs. Among the 172 target genes, 111 genes were annotated by GO database and 69 genes were annotated by KEGG database. The predicted target genes were classified and enriched via GO or KEGG analysis. GO enrichment analysis demonstrated that the target genes of DE miRNAs were divided into three categories: biological process, cellular component, and molecular function. In detail, these genes were involved in many biological processes, and the two most enriched categories were single-organism process and metabolic process (Fig. 5). Interestingly, the GO enrichment analysis of differently expressed mRNAs between big-size group and small-size group of *Siniperca chuatsi* showed that the most enriched categories of biological processes was metabolic process. It indicated that metabolic process might play an important role in growth of *Siniperca chuatsi*.

**Fig. 6:**
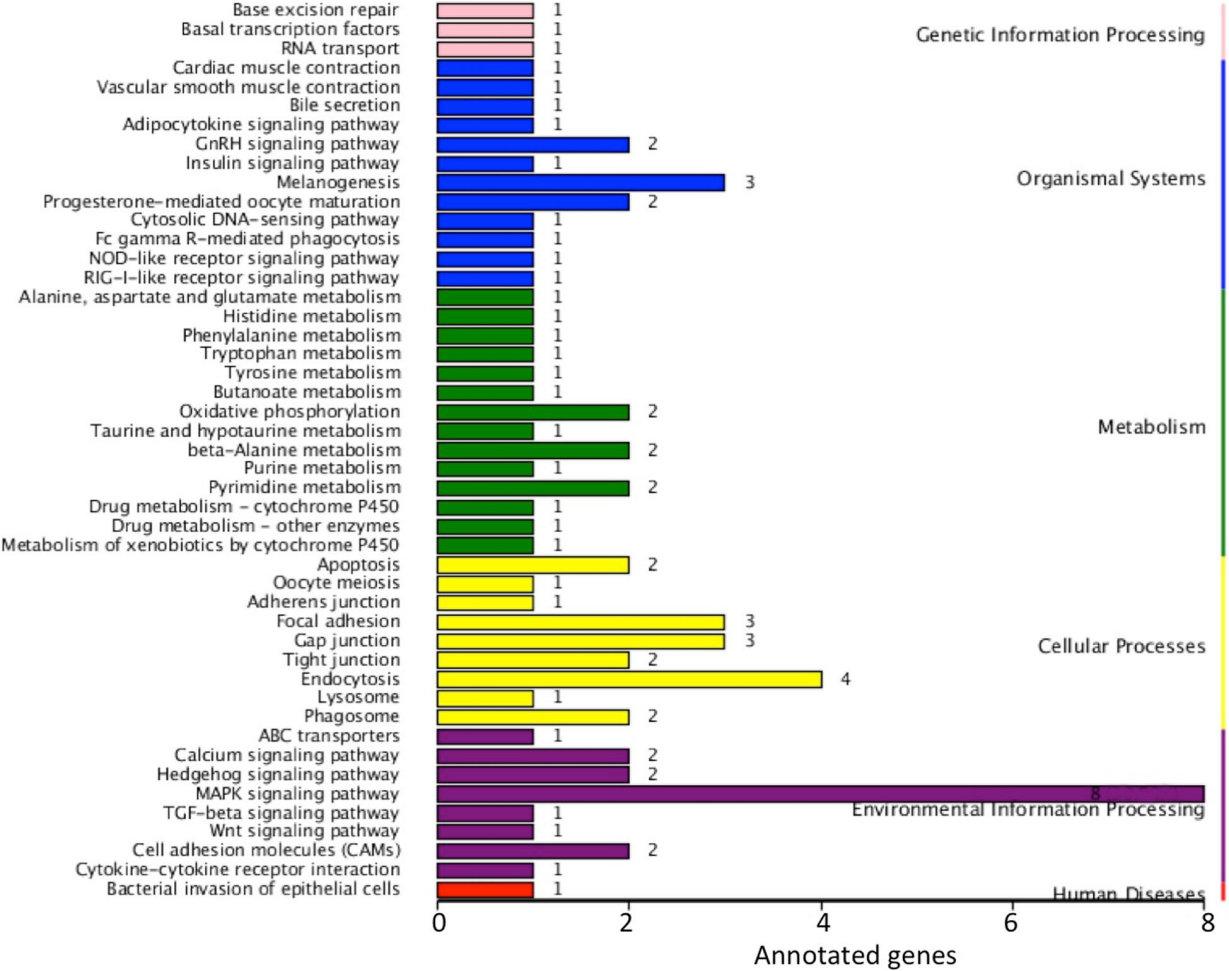
KEGG enrichment analysis of target genes of DE miRNAs between big-size group and small-size group

KEGG analysis showed that enriched target genes were also involved in several metabolic processes (Fig. 6). In addition, we found that the most enriched categories of the target genes belonged to the Mitogen-activated protein kinase (MAPK) signaling pathway (Fig. 6). MAPK signaling pathway has been reported to be highly associated with the growth of cancer cells [34, 35]. Therefore, whether the DE miRNAs observed in this study was associated with growth of *Siniperca chuatsi* via regulating the MAPK signal pathway is worthy of further study.

## Conclusion

In summary, we identified 252 known miRNAs and 12 novel miRNAs from growth-related tissues (brain, pituitary, liver, and muscle) of *Siniperca chuatsi* using Solexa sequencing. Our study provides the first characterization of growth-related miRNAs in *Siniperca chuatsi*. Thirty-one known miRNAs showed differential expression between the big-size and small-size groups of *Siniperca chuatsi*. Functional annotation of the predicted target genes of the DE miRNAs revealed a broad range of the metabolic pathway and biosynthesis processes. These findings support the hypothesis that certain miRNAs might be essential in the regulation of growth, and it will be critical to develop new strategies for the molecular breeding of *Siniperca chuatsi*.

## Additional file

Additional file 1: Target gene prediction and analysis of miRNAs. (XLS 1498 kb)

## Abbreviations

DMTF1: Cyclin-D binding myb-like transcription fector 1
FDR: False discovery rate
GO: Gene Ontology
KEGG: Kyoto Encyclopedia of Genes and Genomes
MAPK: Mitogen-activated protein kinase
MiRNAs: MicroRNAs
qRT-PCR: Quantitative reverse transcription polymerase chain reaction
SOAP: Short Oligonucleotide Analysis Package
TPM: Transcript per million
UTRs: Untranslated regions
